# Perceived satisfaction with HIV care and its impact on medication adherence and clinical outcomes: a cross-sectional study in Kumasi, Ghana

**DOI:** 10.1186/s12879-025-12413-0

**Published:** 2026-01-09

**Authors:** Anthony Kwame Enimil, Nana Kwame Ayisi-Boateng, Solomon Abutiate, Alfred Effah, Samuel Kwarteng, Ebenezer Senu, Stephen Opoku, Success Acheampomaa Oppong, Kingsley Takyi Yeboah, Augustina Lamptey, Mohammed Arafat, Festus Nana Afari-Gyan, Samuel Kwame Sopuruchi Agomuo, Samuel Kekeli Agordzo, Oscar Simon Olympio Mensah, Solomon Akpobi, Tonnies Abeku Buckman, Benjamin Amoani, Samuel Asamoah Sakyi

**Affiliations:** 1 Pediatric Infectious Disease Unit, Child Health Directorate, Komfo Anokye Teaching Hospital, Kumasi, Ashanti region Ghana; 2 Department of Medicine, School of Medical Sciences, Kwame Nkrumah University of Science and Technology, Kumasi, Ghana; 3 Department of Molecular Medicine, School of Medical Sciences, Kwame Nkrumah University of Science and Technology, Kumasi, Ashanti region Ghana; 4 Department of Medical Diagnostics, Faculty of Allied Health Sciences, Kwame Nkrumah University of Science and Technology, Kumasi, Ashanti region Ghana; 5 Laboratory Department, School of Public Health, KNUST-IVI Collaboration Center, Kwame Nkrumah University of Science and Technology, Kumasi, Ashanti region Ghana; 6 Department of Medical Laboratory Sciences, KAAF University College, Buduburam, Accra, Greater Accra region Ghana; 7 Department of Biomedical Science, School of Allied Health Sciences, University of Cape Coast, Cape Coast, Central region Ghana

**Keywords:** HIV/AIDS, HIV/AIDS patients care, PLWH, Medication adherence, Clinical outcomes

## Abstract

**Background:**

HIV/AIDS continues to pose a significant public health challenge, affecting millions of people worldwide. In Ghana, HIV affects 1.5% of the population, totaling 330,000 individuals and resulting in more than 14,000 deaths annually. To date, HIV has no cure and remains a chronic infection, often requiring frequent hospital visits. Given the frequent hospital visits associated with HIV/AIDS, patient satisfaction with care plays a significant role in adherence and treatment outcomes. However, factors associated with satisfaction with care and its impact on clinical outcomes has barely been investigated in the Ghanaian setting. This study assessed the level of satisfaction with care and its association with adherence among people living with HIV (PLWH) in Kumasi, Ghana.

**Methods:**

This cross-sectional study involved 315 PLWH attending the Komfo Anokye Teaching Hospital and Aniniwah Medical Center. A well-structured validated questionnaire was administered to obtain data on sociodemographic, clinical parameters, lifestyle, and satisfaction with care. The Tilburg Frailty Indicator and Adherence to Chronic Disease Scale were used to assess frailty syndrome and medication adherence. Viral load and other relevant medical records were also retrieved from the folders of participants. Statistical analysis was performed using SPSS version 26 and GraphPad Prism version 8.0. *p* < 0.05 was considered statistically significant.

**Results:**

The proportion of participants satisfied with care was 84.1%. Dissatisfaction with care was mainly due to the long waiting time (36.8%) and the lack of logistics (11.4%). The prevalence of high medication adherence, frailty, viral suppression and rebound were 38.7%, 38.4%, 75.5% and 6.6%, respectively. Dissatisfaction with care [aOR = 0.39, 95% CI (0.18–0.85), *p* = 0.017] was independently associated with lower odds of high medication adherence. However, no statistically significant association was observed between satisfaction and frailty.

**Conclusion:**

The level of satisfaction was high among PLWH, with long waiting time and lack of logistics being the main causes of dissatisfaction with care. Satisfaction with care significantly influenced medication adherence, but had no impact on frailty among participants. This calls for measures to reduce patient long waiting time and upgrading logistics at various HIV treatment centers improve patients’ satisfaction and medication adherence.

## Introduction

Human Immunodeficiency Virus/Acquired Immunodeficiency Syndrome (HIV/AIDS) continues to pose a significant public health challenge, affecting millions of people worldwide. Around 38.4 million people worldwide are currently living with HIV, of which 52% reside in sub-Saharan Africa (SSA) making it the most heavily affected region in the world [1–3]. According to UNAIDS, approximately 330,000 individuals in Ghana are living with HIV, representing a prevalence rate of 1.5% among adults aged 15 to 49 [4]. In 2023, there were 18,000 new HIV infections, equating to an incidence rate of 0.56 per 1,000 uninfected individuals [4].

The introduction of combined antiretroviral therapy (cART), previously referred to as Highly Active Antiretroviral Therapy (HAART), has modified the clinical course of HIV infection, reducing the rate of disease progression, the incidence of opportunistic infection and mortality [5]. Adherence to antiretroviral therapy (ART) is vital for achieving viral suppression, reducing the risk of drug resistance, and improving health outcomes. In response, the 95-95-95 initiative was launched in 2020 to ensure that 95% of people living with HIV receive sustained antiretroviral treatment and that 95% of those on treatment achieve viral suppression by 2025 [6].

However, adherence to ART can be challenging despite the availability of more tolerable regimens [7]. In a study conducted within the Kumasi metropolis among people living with HIV (PLWH), approximately 47% did not adhere to their medication [8]. Moreover, studies in regional hospitals in Ghana’s Upper West and Upper East regions reported ART adherence rates of 62.2% and 62.6%, respectively [9], highlighting a high prevalence of suboptimal adherence. Factors such as the complexity of the ART regimen, side effects of the medication, and patients’ perceptions of the quality of care received can impact adherence [10]. One of the most reported determinants of adherence to ARTs is the influence of healthcare workers on patients [1].

Patient satisfaction can be defined as a subjective evaluation of the service received against the individual’s expectations [11]. It is a measure of how well the patient feels their health care needs were met and can be influenced by factors such as communication with health care providers, availability of resources, and timeliness of care. Previous studies have shown that satisfaction with HIV care is associated with improved adherence to antiretroviral therapy (ART) [12], better retention in treatment [13], and ultimately better clinical outcomes for people living with HIV/AIDS. In addition, satisfaction with HIV treatment has been shown to have positive effects on mental health, including lower levels of depression and anxiety [14] and improved quality of life. Understanding the factors that contribute to patient satisfaction with HIV treatment is critical to optimizing health care and improving the overall well-being of those affected by HIV/AIDS. HIV experts and care professionals agree that patient’s participation in satisfaction survey, is an essential part of HIV care and policy making today [15]. Assessing patients’ perceptions of the quality of care not only provides information about the actual experiences but also reveals which quality aspects patients regard as most important [16].

This study assessed the level of satisfaction with care and its association with adherence among people living with HIV (PLWH) in Kumasi, Ghana.

## Methods

### Study design and demography

This was a hospital-based cross-sectional study conducted among People living with HIV in the Ashanti region. The research collaborated with the Komfo-Anokye Teaching Hospital (KATH) and Aniniwah Medical Center’s (AMC) HIV clinics to recruit participants. KATH is the second largest tertiary hospital in Ghana, with a capacity of over 1,200 beds. It functions as a referral center for other hospitals in the central and northern regions of the country. AMC is also located in Kumasi, the capital of the Ashanti region of Ghana and have a well-resourced HIV care clinic making it suitable for the successful implementation of the study.

### Study population and sample size

The sample size was obtained using the Cochran formula $\:n=\:\frac{{z}^{2}pq}{{e}^{2}}$ [17], with *p* as the proportion of satisfaction with HIV care among an African cohort that included Ghana = 89.6% (0.896) [18], q = 1-p, z = z value at 95% confidence (1.96), and e is the margin of error (0.05).

$$n = {{{{1.96}^2}\left({0.896} \right)\left({0.104} \right)} \over {{{0.05}^2}}} = 143.19$$

The minimum sample size required (n) was 143, however, a total of 315 participants comprising 252 from KATH and 63 from the AMC were recruited for this study.

### Inclusion criteria

HIV-positive individuals aged 18 years and above, who were on ART for over 6 months at the HIV clinics of AMC and KATH, from April to November 2023 were recruited.

### Exclusion criteria

HIV-negative individuals, HIV-positive individuals under the age of 18, and newly diagnosed HIV-positive individuals not on ART were excluded from the study.

### Data collection

A well-structured and validated questionnaire was used to collect data from the study participants. The questionnaire consisted of five main sections: sociodemographic information, clinical and lifestyle factors, satisfaction with care, medication adherence, and frailty. The study questionnaire was adapted and modified from previous studies for this research [19–22]. Written consent was obtained from participants before trained research assistants administered the questionnaire, occasionally translating it into local languages to ensure respondents’ understanding.

### Measurement of satisfaction with care

Satisfaction with care was assessed using 9 questions designed to evaluate various aspects of satisfaction with healthcare services. Questions regarding satisfaction were adapted from previous studies and modified for the current study [19, 20]. The questionnaire covered areas such as waiting time, personnel interaction, facility cleanliness, equipment adequacy, and the perceived quality of care received. Each question is structured to elicit a binary response, where positive feedback is assigned a score of 1, indicating satisfaction, while a negative response is scored 0, reflecting dissatisfaction. The overall satisfaction score ranges from 0 to 9. Participants scoring 4 or more were classified as satisfied, while those scoring below 4 were considered not satisfied.

### Measurement of frailty using the tilburg frailty indicator

The Tilburg frailty indicator was developed by Gobbens et al. [21] and consists of two parts. The first part collects the participant’s sociodemographic information, while the second part contains 15 self-reported questions divided into social, psychological and physical categories. The physical domain includes eight questions rated from 0 to 8 points related to physical health, unexplained weight loss, difficulty walking, balance, hearing and vision problems, hand strength, and physical fatigue. The psychological domain consists of four items, each scored 0–4, that focus on cognition, depressive symptoms, anxiety, and coping. The social area includes three questions worth 0–3 points each about social interactions, receiving support and life situations. In the second part, there are usually yes or no answer options; for some questions, there is also the third option “Occasionally”. Each “yes” or “sometimes” answer receives one point, and a “no” answer receives zero points. The total score can range from 0 to 15, with higher scores indicating increased frailty. A total TFI score of 5 or more indicates frailty. The TFI is proven to be a reliable and valid tool for assessing frailty.

### Measurement of medication adherence using Adherence in Chronic Disease Scale (ACDS)

Adherence in Chronic Disease Scale (ACDS) was used to evaluate compliance with medication adherence among study participants. The presumption of ACDS lies in the fact that only high adherence reflects good, therapeutic plan realization in respect to pharmacology. The scale consists of 7 questions with 5 possible answers each. The questions reflect behaviors directly determining adherence (questions 1–5) and the situations and opinions indirectly affecting adherence (questions 6–7). The result of ACDS ranges from 0 to 28 points, with higher scores or points indicating better adherence. The score is interpreted as; above 26 points – high adherence, between 21 and 26 points – moderate adherence, below 21 points – low adherence [22].

### Operational definitions

The results of the first viral load test, conducted 6 months after diagnosis, were collected. Subsequent viral load test results were recorded at 12, 24, 36, and 48 months. Viral suppression was defined as having at least one viral load measurement below 50 copies/mL after starting treatment, whilst viral rebound was defined as having at least one viral load of 50 copies/mL or higher following a period of suppression [23]. Viral loads were taken 6 months apart per treatment guidelines and clinicians reviews.

Satisfaction with care – was defined as a score of 4 or more in the satisfaction with care section, whiles dissatisfaction with care was defined as a score of less than 4 in the satisfaction with care section.

### Statistical analysis

Data analysis was performed using Statistical Package for the Social Sciences 26.0 software (SPSS, Inc.; Chicago, IL, USA) and GraphPad Prism version 8.0. Descriptive statistics (frequency and percentage) were used to summarize variables. The proportion of satisfaction and dissatisfaction with care was illustrated with a simple bar chart. A Pearson chi-square was done to identify the association between Satisfaction with care and the other factors at a significance level of 0.05. Furthermore, Univariate and Multivariate logistic regression models were run on the significant variables from the Chi-square test to determine independent predictors of satisfaction with care. *p*-values less than 0.05 and 95% confidence interval were considered statistically significant.

## Results

### Sociodemographic characteristics of study participants

Of the 315 participants enrolled in the study, one-third (33.3%) were within the ages of 46-55years. Most of the participants in the study were females (85.7%) and 37.5% were married. Also, a higher percentage of the participants had 2–3 children (44.3%) with 39.0% having Junior High School education (39.0%). The majority of the enrolled participants lived in urban areas (85.7%) and were Christians (87.6%). With respect to employment status, 7.3% were employees whilst 63.5% were self-employed. Moreover, 47.5% of the participants earned <₵500 and 45.0% took 31–60 min to reach the hospital clinic (Table 1).

**Table 1:** Sociodemographic characteristics of study participants

Variable	Frequency (*n* = 315)	Percentage (%)
**Age Group (Years)**		
18–35	41	13.0
36–45	80	25.4
46–55	105	33.3
56–90	89	28.3
**Gender**		
Female	270	85.7
Male	45	14.3
**Marital Status**		
Single	55	17.5
Divorced	61	19.4
Widowed	81	25.7
Married	118	37.4
**Number of Children**		
0–1	73	23.2
2–3	139	44.3
4 or more	102	32.5
**Educational Level**		
No formal	64	20.3
Primary	63	20.0
JHS	123	39.0
SHS	49	15.6
Tertiary	16	5.1
**Residence**		
Rural	45	14.3
Urban	270	85.7
**Employment Status**		
Not employed	92	29.2
Self employed	200	63.5
Employed	23	7.3
**Ethnicity**		
Akan	257	81.6
Northerners	46	14.6
Others	12	3.8
**Religion**		
Christianity	275	87.5
Muslims	36	11.5
Others	3	1.0
**Monthly Income (GH**₵**)**		
< 500	149	47.5
500–1000	107	34.0
> 1000	58	18.5
**Time taken to reach hospital (minutes)**		
5 to 30	74	23.6
31 to 60	141	45.1
More than 60	98	31.3

Data presented as frequency and percentage. JHS; Junior High School, SHS: Senior High School. Monthly income in dollar ($); <$32, $32–65, >$65

### Clinical and lifestyle characteristics of study participants

Of the 315 participants recruited, more than half (56.8%) take fruits occasionally, 66% had experienced no frequent memory hitches and 69.4% undertook physical exercises. Approximately three-quarters (72%) of these had no other chronic medical conditions for which they were taking drugs **(**Table 2**)**.

**Table 2:** Clinical and lifestyle characteristics of study participants

Variable	Frequency(*n* = 315)	Percentage (%)
**Problems with memory**		
No	208	66.0
Yes	107	34.0
**How often fruit is taken**		
Daily	52	16.5
Weekly	84	26.7
Occasionally	179	56.8
**Other medical conditions**		
No	226	72.0
Yes	88	28.0
**Physical Exercise**		
No	96	30.6
Yes	218	69.4

Data presented as frequency and percentage

### Responses to satisfaction with HIV care and proportion of satisfaction level

Of the 315 participants interviewed, waiting time recorded the highest source of dissatisfaction with care (36.8%) followed by quality of clinic building infrastructure and logistics (11.4%). Cleanliness of the clinic recorded the highest source of satisfaction of 97.8% also followed closely by confidentiality of the healthcare providers (96.5%) (Table 3).

**Table 3:** Responses to satisfaction with HIV care

Variable	Frequency (*n* = 315)	Percentage
**Waiting time**		
Not satisfied	116	36.8
Satisfied	199	63.2
**Competence and skills of healthcare workers**		
No	17	5.4
Yes	298	94.6
**Attitudes of healthcare workers**		
Not satisfied	30	9.5
Satisfied	285	90.5
**Quality of clinic building infrastructure and logistics**		
No	36	11.4
Yes	279	88.6
**Confidentiality of healthcare workers**		
Unsatisfactory	11	3.5
Satisfactory	304	96.5
**Cleanliness of clinic**		
No	7	2.2
Yes	308	97.8
**Support system and counselling**		
No	15	4.8
Yes	300	95.2
**Would you recommend the clinic**		
No	12	3.8
Yes	303	96.2
**Rate overall quality**		
Unsatisfactory	12	3.8
Satisfactory	303	96.2

Data presented as frequency and percentage

### Proportion of satisfaction level

**Fig. 1:**
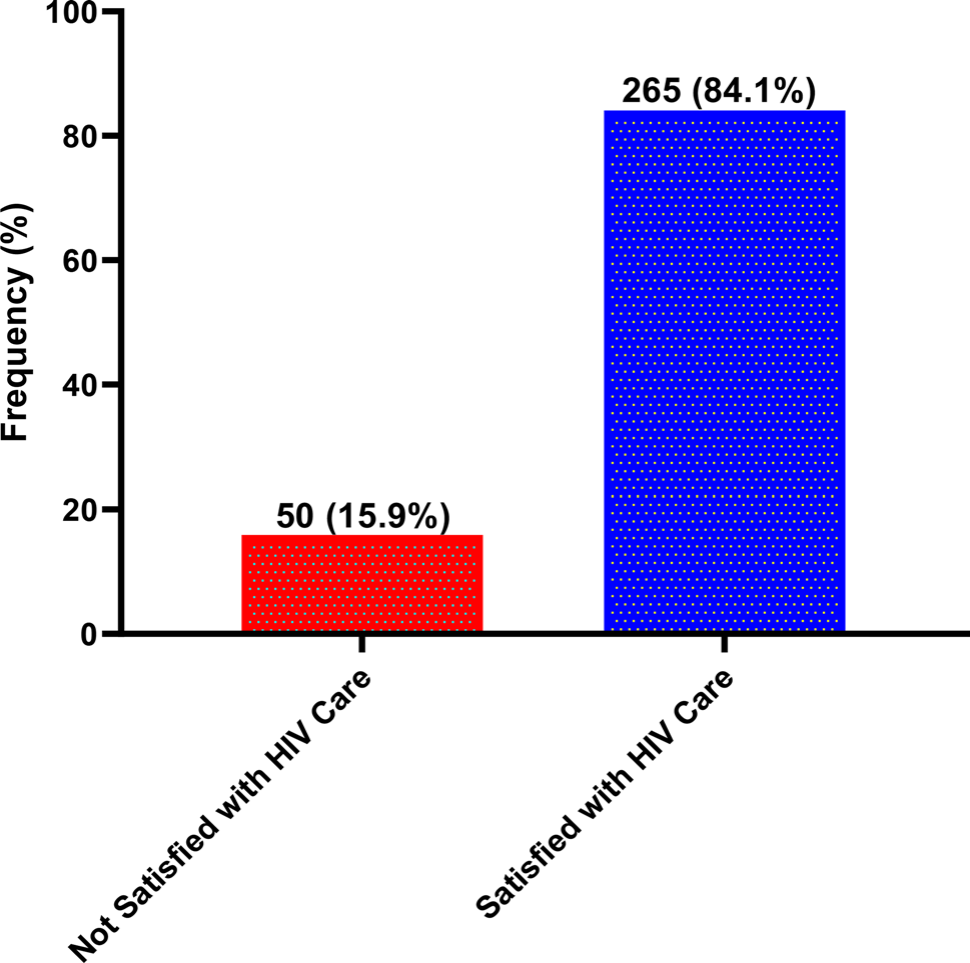
Proportion of satisfaction with care among PLWH

Of the 315 participants recruited, 84.1% were satisfied with the care they received. Satisfaction was defined as a score of seven and above (≥ 4) after grading participants’ response (Fig. 1).

### Characteristics of clinical outcomes among people living with HIV on treatment

Analysis of the clinical outcome parameters showed that some participants were not adhering enough to their medications (7.6%), most patients adhered moderately (53.7%) and 38.7% recorded high adherence. Approximately 38.4% of the participants were frail. One-third of the participants (75.5%) had suppressed viral load and most of them (93.4%) had no rebound (Table 4).

**Table 4:** Characteristics of clinical outcomes among people living with HIV on treatment

Variable	Frequency (*n* = 315)	Percentage (%)
**Adherence**		
Low adherence	24	7.6
Moderate	169	53.7
High adherence	122	38.7
**Suppression**		
Not suppressed	70	24.5
Suppressed	216	75.5
**Rebound**		
Not rebound	267	93.4
Rebound	19	6.6
**Frailty**		
Not frail	194	61.6
Frail	121	38.4

Data presented as frequency and percentage

### Predictors of medication adherence among PLWH

In a univariate logistic regression model, being aged 36–45 years, 46–55 years, residing in rural areas, having no formal, primary, or junior high school (JHS) education, being single, and reporting unsatisfaction with HIV care were significantly associated with lower odds of high medication adherence among PLWH.

After adjusting for possible confounders in a multivariate logistic regression model, being aged 36–45 years [aOR = 0.36, 95% CI (0.15–0.86), *p* = 0.022] or 46–55 years [aOR = 0.35, 95% CI (0.15–0.87), *p* = 0.023], residing in a rural area [aOR = 0.34, 95% CI (0.15–0.76), *p* = 0.009], being single [aOR = 0.44, 95% CI (0.20–0.99), *p* = 0.046], consuming alcohol [aOR = 0.42, 95% CI (0.18–0.95), *p* = 0.037], and being dissatisfied with care [aOR = 0.39, 95% CI (0.18–0.85), *p* = 0.017] were independently associated with lower odds of high medication adherence (Table 5).

**Table 5:** Univariate and multivariate predictors of high adherence to medication among study participants

Variable	High adherence (*n* = 122)	cOR (95% CI)	*p*-value	aOR (95% CI)	*p*-value
**Age Group (Years)**					
18–35 (Ref)	23 (18.9)	1.00	-	1.00	-
36–45	27 (22.1)	0.40 (0.18–0.86)	**0.019**	0.36 (0.15–0.86)	**0.022**
46–55	36 (29.5)	0.41 (0.20–0.85)	**0.017**	0.35 (0.15–0.87)	**0.023**
56–90	36 (29.5)	0.53 (0.25–1.12)	0.098	0.46 (0.18–1.17)	0.460
**Gender**					
Female	100 (82.0)	0.62 (0.33–1.16)	0.133	0.62 (0.29–1.32)	0.211
Male (Ref)	22 (18.0)	1.00	-	1.00	-
**Marital Status**					
Single	18 (14.8)	0.64 (0.33–1.25)	0.191	0.44 (0.20–0.99)	**0.046**
Divorced	20 (16.4)	0.64 (0.34–1.22)	0.178	0.69 (0.34–1.40)	0.301
Widowed	33 (27.0)	0.90 (0.51–1.60)	0.728	0.99 (0.51–1.95)	0.997
Married (Ref)	51 (41.8)	1.00	-	1.00	-
**Educational Level**					
No formal	22 (18.0)	0.24 (0.07–0.77)	**0.017**	0.29 (0.07–1.21)	0.088
Primary	24 (19.7)	0.28 (0.09–0.90)	**0.033**	0.29 (0.07–1.18)	0.083
JHS	45 (36.9)	0.26 (0.09–0.80)	**0.019**	0.27 (0.07–1.03)	0.055
SHS	20 (16.4)	0.31 (0.09–1.04)	0.058	0.29 (0.07–1.16)	0.080
Tertiary (Ref)	11 (9.0)	1.00	-	1.00	-
**Residence**					
Rural	9 (7.4)	0.35 (0.16–0.75)	**0.007**	0.34 (0.15–0.76)	**0.009**
Urban (Ref)	113 (92.6)	1.00	-	1.00	-
**Employment Status**					
Not employed (Ref)	35 (28.7)	1.00	-	1.00	-
Self employed	76 (62.3)	0.99 (0.60–1.66)	0.994	0.85 (0.47–1.54)	0.583
Employed	11 (9.0)	1.49 (0.59–3.75)	0.393	0.69 (0.21–2.25)	0.534
**Monthly Income (GH**₵**)**					
< 500	61 (50.0)	0.85 (0.46–1.57)	0.853	1.08 (0.47–1.54)	0.583
500–1000	35 (28.7)	0.60 (0.31–1.15)	0.125	0.83 (0.39–1.76)	0.618
> 1000 (Ref)	26 (21.3)	1.00	-	1.00	-
**Alcohol intake**					
No (Ref)	111 (91.0)	1.00	-	1.00	-
Yes	11 (9.0)	0.52 (0.25–1.07)	0.077	0.42 (0.18–0.95)	**0.037**
**Satisfaction with HIV care**					
Not satisfied	11 (9.0)	0.39 (0.19–0.80)	**0.010**	0.39 (0.18–0.85)	**0.017**
Satisfied (Ref)	111 (91.0)	1.00	-	1.00	-

Note: cOR: crude odds ratio, aOR: adjusted odds ratio, CI: Confidence interval, *p* < 0.05 and bolded means statistically significant

### Predictors of frailty among PLWH

In a univariate logistic regression model, being widowed [cOR = 2.14, 95% CI (1.19–3.83), *p* = 0.011], having no formal education [cOR = 4.33, 95% CI (1.13–16.68), *p* = 0.033] and being unsatisfied with HIV care [cOR = 1.94, 95% CI (1.05–3.56), *p* = 0.033] were associated with higher odds of being frail. However, being self-employed [cOR = 0.39, 95% CI (0.23–0.64), *p* < 0.001] or employed [cOR = 0.22, 95% CI (0.08–0.65), *p* = 0.006], engaging in exercise [cOR = 0.50, 95% CI (0.31–0.82), *p* = 0.006], taking fruits daily [cOR = 0.49, 95% CI (0.25–0.96), *p* = 0.037] or weekly [cOR = 0.51, 95% CI (0.30–0.89), *p* = 0.018], and having moderate [cOR = 0.17, 95% CI (0.06–0.47), *p* = 0.001] or high adherence [cOR = 0.11, 95% CI (0.04–0.32), *p* < 0.001] were significantly associated with lower odds of frailty among PLWH.

After adjusting for potential confounders in a multivariate logistic regression model, being self-employed [aOR = 0.45, 95% CI (0.24–0.83), *p* = 0.011], having moderate adherence to ART [aOR = 0.21, 95% CI (0.07–0.68), *p* = 0.009], and having high adherence [aOR = 0.15, 95% CI (0.05–0.49), *p* = 0.002] remained independently associated with significantly lower odds of frailty among PLWH (Table 6).

**Table 6:** Univariate and multivariate predictors of frailty among study participants

Variable	Frail (*n* = 121)	cOR (95% CI)	*p*-value	aOR (95% CI)	*p*-value
**Age Group (Years)**					
18–35 (Ref)	11 (9.1)	1.00	-	1.00	-
36–45	26 (21.5)	1.31 (0.57–3.02)	0.522	1.32 (0.51–3.43)	0.575
46–55	46 (38.0)	2.12 (0.96–4.69)	0.062	1.88 (0.70–4.99)	0.209
56–90	38 (31.4)	2.03 (0.90–4.56)	0.086	1.70 (0.61–4.72)	0.312
**Gender**					
Female	109 (90.1)	1.86 (0.92–3.76)	0.084	1.66 (0.69–4.04)	0.260
Male (Ref)	12 (9.9)	1.00	-	1.00	-
**Marital Status**					
Single	21 (17.4)	1.35 (0.69–2.64)	0.377	1.26 (0.57–2.81)	0.567
Divorced	23 (19.0)	1.33 (0.69–2.53)	0.394	1.07 (0.51–2.22)	0.867
Widowed	40 (33.1)	2.14 (1.19–3.83)	**0.011**	1.65 (0.81–3.34)	0.165
Married (Ref)	37 (30.6)	1.00	-	1.00	-
**Educational Level**					
No formal	32 (26.4)	4.33 (1.13–16.68)	**0.033**	2.66 (0.52–13.73)	0.242
Primary	24 (19.8)	2.67 (0.69–10.33)	0.156	1.78 (0.35–9.03)	0.488
JHS	47 (38.8)	2.68 (0.73–9.90)	0.139	1.71 (0.35–8.33)	0.505
SHS	15 (12.4)	1.91 (0.47–7.71)	0.362	1.24 (0.25–6.25)	0.794
Tertiary (Ref)	3 (2.5)	1.00	-	1.00	-
**Residence**					
Rural	19 (15.7)	1.20 (0.63–2.28)	0.571	0.80 (0.38–1.67)	0.546
Urban (Ref)	102 (84.3)	1.00	-	1.00	-
**Employment Status**					
Not employed (Ref)	51 (42.1)	1.00	-	1.00	-
Self employed	65 (53.7)	0.39 (0.23–0.64)	**< 0.001**	0.45 (0.24–0.83)	**0.011**
Employed	5 (4.1)	0.22 (0.08–0.65)	**0.006**	0.42 (0.12–1.53)	0.189
**Monthly Income (GH**₵**)**					
< 500	60 (50.0)	1.38 (0.73–2.62)	0.319	0.73 (0.34–1.57)	0.416
500–1000	41 (34.2)	1.28 (0.65–2.50)	0.479	0.84 (0.39–1.82)	0.656
> 1000 (Ref)	19 (15.8)	1.00	-	1.00	-
**Alcohol intake**					
No (Ref)	100 (82.6)	1.00	-	1.00	-
Yes	21 (17.4)	1.73 (0.90–3.32)	0.100	1.66 (0.75–3.69)	0.215
**Smoking**					
No (Ref)	117 (97.5)	1.00	-	1.00	-
Yes	3 (2.5)	1.63 (0.32–8.22)	0.552	1.83 (0.27–12.46)	0.537
**Exercise**					
No (Ref)	48 (39.7)	1.00	-	1.00	-
Yes	73 (60.3)	0.50 (0.31–0.82)	**0.006**	0.72 (0.41–1.25)	0.244
**Frequency of fruit intake**					
Daily	15 (12.4)	0.49 (0.25–0.96)	**0.037**	0.71 (0.33–1.51)	0.370
Weekly	25 (20.7)	0.51 (0.30–0.89)	**0.018**	0.76 (0.41–1.42)	0.386
Occasionally (Ref)	81 (66.9)	1.00	-	1.00	-
**Satisfaction with HIV care**					
Not satisfied	26 (21.5)	1.94 (1.05–3.56)	**0.033**	1.37 (0.67–2.81)	0.386
Satisfied (Ref)	95 (78.5)	1.00	-	1.00	-
**Adherence**					
Low (Ref)	19 (15.7)	1.00	-	1.00	-
Moderate	66 (54.5)	0.17 (0.06–0.47)	**0.001**	0.21 (0.07–0.68)	**0.009**
High	36 (29.8)	0.11 (0.04–0.32)	**< 0.001**	0.15 (0.05–0.49)	**0.002**

Note: cOR: crude odds ratio, aOR: adjusted odds ratio, CI: Confidence interval, *p* < 0.05 and bolded means statistically significant

## Discussion

Patient satisfaction with care is an important factor that can impact on the quality of care. As it influences long-term adherence and retention in care, patient satisfaction can have a significant impact on healthcare outcomes. When satisfied with care, patients may trust their healthcare providers and stick to all instructions given. Nonetheless, to better satisfy patients, it is essential that healthcare providers identify factors patients deem important in their care. In this study, the proportion of participants satisfied with their care was 84.1%. Of the participants dissatisfied with care, waiting time was found to be the predominant factor followed by a lack of adequate clinic logistics. It was further observed that satisfaction with care was significantly associated with adherence to medication. There was no significant association between satisfaction with care and frailty.

The proportion of participants satisfied with care in this study was 84.1%, which aligns with findings from previous studies. Gezahegn et al. (2021) reported a satisfaction rate of 85.5% in Ethiopia, while the African Cohort Study (AFRICOS) conducted across Tanzania, Kenya, Uganda, and Nigeria by Somi et al. (2021) found a satisfaction rate of 89.6% [24, 25]. These similarities suggest a consistently high level of patient satisfaction with HIV care across different African settings. However, Wung et al., (2016) reported a significantly high satisfaction with care level of 91.2% among PLWH at the Bamenda Regional Hospital, compared to this study’s findings [26]. Another study by Umeokonkwo et al., (2018) in Nigeria also observed satisfaction levels of 71.5% and 41.4% for public and private hospitals respectively [27] which is lower compared to the current findings. The differences in findings could be attributed to variations in sample sizes and geographical locations.

Our study also demonstrated that most of the respondents were satisfied with the cleanliness of the clinic and the confidentiality of the healthcare providers. On the other hand, the predominant reason for dissatisfaction with care was the waiting time at the clinic. Studies conducted in Dar es Salam, Jimma town and Kenya [24, 25, 28] all had similar results stating waiting time as the main culprit in dissatisfaction with care. A study conducted by Huang et al. showed that when patients arrive on schedule and wait no more than 37 min, they typically seem to be reasonably satisfied [29]. The probable reasons for long waiting times could be the large number of patients at the clinic, the late opening of the clinic on each day or inadequate staff. Moreover, patients will inevitably have to wait longer because there are virtually no computer systems in place to handle the enormous patient load, particularly in record keeping and manual retrieval of records. To deal with the long waiting times, it has been suggested that clinics employ more staff or improve facilities such as seating and refreshments to make waiting more bearable [26, 30, 31].

In the current study, 53.7% of participants demonstrated moderate adherence, while 38.7% exhibited high adherence. These results align with findings from studies conducted in Ghana, which reported ART adherence rates of 44.6% and 47.5% [32, 33]. Similarly, Afe and Motunrayo, (2017) observed 42.0% adherence, which is consistent with our findings [34]. In contrast, Tchakoute et al., (2022). reported a mean adherence level of 84.9%, which is higher compared to our finding [35]. The differences in findings could be explained by variations in how medication adherence was assessed. The current study utilized the Adherence in Chronic Medication Scale, which evaluates adherence at a single point in time, whereas Tchakoute and colleagues applied the Medication Possession Ratio, which measures adherence over a specific period. While the single-point-in-time assessment provides a snapshot of adherence behavior at the moment of data collection, it may not fully capture long-term adherence patterns. Moreover, adherence in this study was assessed based on participant responses during the survey, which may introduce recall bias. Future studies may benefit from incorporating longitudinal adherence measures to provide a more comprehensive understanding of adherence trends over time. In a similar study conducted in Brazil, moderate adherence was 44.3% whilst strict high adherence was practiced among 13.5% of the total study population all of which are low compared to our results [36]. These differences could be attributed to variations in geographical location and sample size.

Viral suppression and rebound were seen in about 75.5% and 6.6% of the study participants. A similar study conducted in Kumasi, Ghana got a consistent viral suppression rate of 76.1% but a higher rebound of 21.0% [37]. The observed viral suppression rate in our study is similar but slightly higher than the national statistics(68%) [38]. Again, when compared to our findings, Lokpo et al., (2020) reported a lower adherence level of 69% in the volta region of Ghana, highlighting the need for policy makers to institute measures to achieve the 95-95-95 target by 2025 [39]. The main challenges to viral suppression identified in Ghana include stigma, discrimination, abandoning of treatment for prayer camps and false claims of cure [40]. In contrast to the sub-optimal viral suppression rates in Ghana, an African cohort study by Somi et al., (2021) reported a high viral suppression rate of 87.5% [24]. This shows that other parts of the continent are on track.

Our study findings showed a significant association between satisfaction with care and adherence to medication. Being dissatisfied with HIV decreased the likelihood of high medication adherence. These results are comparable with works like that performed by Gezahegn et al. who found satisfaction with care to be associated with adherence to medication [25]. Somi et al. also reported the association between satisfaction with care and adherence and further observed no significant association between satisfaction and viral suppression [24]. This finding highlights the need for patient-centered care and the health care experience in enabling ideal treatment outcomes. Moreover, the current finding emphasizes the importance of improving communication, trust, and the general health care environment to foster adherence behaviors in PLWH. Being responsive to patients’ concerns, delivering respectful care, and developing good provider-patient relationships could appreciably improve adherence, reduce the risk of viral rebound, and ultimately result in better long-term virologic and immunologic outcomes.

Our study offers valuable insights to guide HIV clinicians and policymakers in combating the infection. However, it has certain limitations, we relied on existing hospital records for patients’ viral load results, which posed challenges in terms of data completeness. Although the sample size was adequate for the study, we were unable to establish significant associations between satisfaction and other study variables such as age, educational status and income level. Moreover, some variables that could have provided additional context, such as comorbidities, were not included due to data unavailability. A larger sample size and more comprehensive data collection would be necessary to strengthen future findings.

## Conclusion

The level of satisfaction was high among PLWH, with long waiting time and lack of logistics being the main causes of dissatisfaction with care. Satisfaction with care significantly influenced medication adherence, but had no impact on frailty among participants. This calls for measures to upgrade logistics such as electronic medical records systems and appointment scheduling systems to reduce patient waiting time. Implementing these improvements at HIV treatment centers can enhance patient satisfaction and medication adherence.

## Data Availability

All data generated or analyzed during this study are included in this article and can be requested from the corresponding author.

## Abbreviations

HIV/AIDS: Human Immunodeficiency Virus/ Acquired Immune Deficiency Syndrome (AIDS)
PLWH: People living with HIV
TB: Tuberculosis
CHRPE: Committee on Human Research, Publication, and Ethics
KNUST: Kwame Nkrumah University of Science and Technology
WHO: World Health Organization
